# Assessing social values for California's efforts to reduce the overuse of unnecessary medical care

**DOI:** 10.1111/hex.12644

**Published:** 2017-11-16

**Authors:** Susan L. Perez, Desiree Backman, Marge Ginsburg

**Affiliations:** ^1^ Department of Kinesiology and Health Science California State University Sacramento CA USA; ^2^ California Department of Health Care Services and University of California Davis Institute for Population Health Improvement Sacramento CA USA; ^3^ University California Davis Medical Center California Department of Health Care Services Sacramento CA USA; ^4^ Center for Healthcare Decisions Sacramento CA USA

**Keywords:** deliberation, health decisions, medical evidence, overuse

## Abstract

**Background:**

A partnership of large health‐care purchasers created a workgroup to reduce the overuse of harmful and wasteful medical care in California.

**Objective:**

Employ a civic engagement process to identify the social values important to the public in considering different strategies to reduce overuse.

**Intervention:**

Use of deliberation techniques for 3 case examples that explore possible strategies: physician oversight, physician compensation, increased patient cost‐sharing or taking no definitive action.

**Results:**

Five themes were identified, including strong support for physicians’ leadership role to reduce overuse; nuanced enthusiasm for increasing patient cost‐sharing to discourage excessive demand; and marked disapproval of physician compensation as a motivator.

**Conclusion:**

Most but not all of the perspectives voiced by participants are congruent with efforts to reduce overuse that is being initiated or discussed at the state, provider and health plan level. As health‐care policymakers and leaders consider more targeted approaches to reducing overuse, these findings will inform decision‐making.

## 1 INTRODUCTION

The excessive use of unwarranted medical care has concerned health‐care and policy leaders for many years. Policy experts contend that up to 30% of health‐care dollars in the United States is inefficiently used, and a good proportion is linked to the overuse of unnecessary, harmful and/or wasteful medical care.^1^, ^2^, ^3^ Health advocates increasingly aim to engage and activate consumers and communities in improving the health and health care of individuals and populations.^4^ A significant aspect of this engagement is the use of medical evidence to inform and motivate actions that promote high‐quality, affordable care.^5^ Approaches for reducing overuse often reduce access to certain medical services, which may be alarming to those who equate more care with better care or are concerned about interference with doctor–patient decisions. The evolving health‐care landscape calls for greater participation by the public to help specify the principles for attaining high‐quality care as well as responsible use of resources.^6^

Social values assessment is distinct from patient preferences. Most patient decisions concern the clinical care their physicians recommend, such as tests, treatments or procedures. The factors patients consider in deciding to accept or reject recommended individual care are an important aspect of patient‐centered research.^7^ However, when individuals are asked as citizens to consider broader health‐care policy issues in which many people will be affected, their focus expands beyond their personal interests to weighing the economic, social and ethical impacts on the larger community in terms of collective, societal values.^8^

Potential strategies to reduce overuse may threaten aspects of health‐care delivery that the public highly values, such as physician autonomy or patient choice. Physicians’ central role in the prevalence of overuse^9^ suggests that patients’ implicit trust in physician expertise may sometimes be misplaced. This discrepancy prompts such questions as: What types of strategies to reduce overuse, if any, does the public support and for what reasons? Does the public trust research indicating that many physicians are not delivering high‐quality care? Will they support strategies that limit physician autonomy? Are there circumstances where the best interests of society should trump the desires of individual patients?

Among efforts to reduce the frequency of overuse, the 3 largest purchasers in California—the California Department of Health Care Services (the administrator for Medi‐Cal, the state's public insurance programme), California Public Employees Retirement System (state agency, CalPERS, that manages pension and health benefits for California public employees) and Covered California (the state's health benefit exchange established by the Federal Affordable Care Act where individuals can purchase low‐cost health insurance)—joined forces in 2015 to identify statewide priorities and establish processes that will help reduce unnecessary care. This public‐private partnership, called Smart Care California, involves coverage for more than 16 million Californians and aims to meaningfully reduce the level of inappropriate care over a 2‐year period.^10^ Smart Care California's overarching plan includes a deliberative process to capture the views of the lay public.^10^ While California has long demonstrated efforts to identify the public's perspective on challenging health policy issues,^11^, ^12^ this was the first time that state policy leaders incorporated a social values assessment into their work plan.

Consequently, as part of this partnership, the Center for Health‐care Decisions (CHCD)—a non‐profit specializing in civic engagement in health‐care policy—developed *Doing What Works (DWW)*, using a non‐partisan, carefully structured qualitative process called public deliberation.^13^ This article identifies themes resulting from *DWW* that are central to the public's views on reducing overuse of medical care and illustrates how these themes are consistent with or diverge from existing and proposed health‐care policy.

## 2 METHOD

### 2.1 Methods design

This deliberative process asked the public to help address the problem of overuse by recognizing that they are responding as citizen decision makers providing input to policy decisions; considering the impact of overuse on society at large; and debating why various resolutions to the problem are more acceptable than others.^6^

### 2.2 Recruitment and sample

Experienced, non‐partisan facilitators led 10 DWW sessions, each lasting 4 and a half hours, in 6 communities, urban and rural, across California. Five groups consisted of Medi‐Cal members; 4 groups included individuals who purchased insurance through Covered California; and one group of individuals was insured through CalPERS. These groups were chosen because they are insured members of the project sponsors. To establish commonality among the groups, all participants would be low‐to‐moderate income individuals, those most likely to be sensitive to higher cost‐sharing or reduced coverage.^1^

Professional recruitment companies and community‐based organizations recruited the 117 participants. Inclusion criteria were limited to individuals between the ages of 30 and 60 years to capture the perspectives of those who are more likely to have had experience interacting with their health‐care system and are not on Medicare. Each participant received a $200 incentive to participate. Table 1 shows the demographics of the participants.

**Table 1:** Doing What Works participant demographics

Category	Study participants (N = 117)
Gender	
Male	36%
Female	64%
Insurer	
Medi‐Cal (Medicaid)	51%
Covered California	38%
CalPERS	10%
Education	
Not a high school graduate	3%
High school graduate	32%
Some college	29%
Associate of arts degree	8%
College graduate	23%
Post‐graduate	6%
Race/Ethnicity	
Latino/Hispanic	40%
Black people/African American	10%
White people/Anglo	41%
Asian/Pacific Islander	5%
Other	3%

Authors’ analysis of data from Doing What Works participant surveys, 2015.

### 2.3 Ethics

The California State University, Sacramento Institutional Review Board approved this project (IRB 14‐15‐126).

### 2.4 Data collection

During each session, participants completed a pre‐ and post‐survey to capture demographic data as well as assess and determine shifts in beliefs and attitudes regarding the use of evidence to inform treatment and coverage decisions pertaining to low‐value care. Pre‐ and post‐survey results can be found in the DWW Final Report.^14^

Each session commenced with a review and discussion of an educational handout describing the problem of medical care overuse; the meaning of high‐ and low‐value care; the reasons low‐value care exists and its harms; and the medical research that forms the basis for evidence‐based practice. The educational handout was developed with input from the DWW Advisory Committee, which was comprised of consumer advocates, health education experts, a patient representative and health‐care policy leaders.

Participants then reviewed and discussed in turn 3 case scenarios illustrating the problem of overuse: antibiotics for adult bronchitis; Caesarean sections for low‐risk births; and Magnetic Resonance Imaging (MRI) for assessment of acute low‐back pain (see Supporting Information A). Each case scenario included utilization data, medical harms and costs of overuse based on California or national figures. A multidisciplinary advisory committee provided input to assure the accuracy and impartiality of the educational materials, case scenarios and facilitator's guide. The case scenarios were reviewed by the DWW Advisory Committee to ensure accuracy of information and were assessed for literacy at a 7th‐ to 8th‐grade reading level using the Flesch Reading Ease Scale.

The 3 case scenario topics were chosen in consultation with policy leaders and an assessment of existing statewide efforts. For a successful deliberative process, a number of criteria must be met as follows: (i) there is a range of policy options to be considered from gentle to draconian; (ii) there are tensions among values (eg, patient autonomy, trust, harms to others, responsibility, resource use); and (iii) the topic must be easily relatable to participants, avoiding complex case examples that are difficult for the public to grasp.^6^

For each case scenario, participants considered 5 strategies or potential approaches to reducing overuse: (i) establish greater oversight and control of physicians who overuse (monitoring physicians); (ii) reduce demand by increasing patient cost‐sharing for unnecessary care; (iii) influence physicians through rewards; (iv) influence physicians through non‐payment; and (v) take no specific action, therefore continuing to leave these decisions to individual physicians and their patients.^15^ Participants were then instructed to choose the most acceptable strategy to reduce overuse. On a visible flipchart, the co‐facilitator wrote down each participant's selection. Participants then discussed and debated their choices; some people changed their mind or endorsed additional strategies during the discussion.

### 2.5 Data analysis

Quantitatively, the number and percentage of participants who favoured the various strategies were central to assessing results. But the rationale for their choices was determined through qualitative analysis. Discussions were audio‐recorded, transcribed and analyzed using inductive, grounded theory methodology. Transcript analysis focused on the reasons, values and rationale given by participants for why certain strategies were more or less desirable than others and how the different scenarios influenced their perspective. The development of themes was theoretically guided by the constant comparison method: (i) immersion in the transcripts (reading and rereading); (ii) the development of themes and codes; (iii) coding the transcripts; and (iv) reintegrating the codes into an explanatory narrative. Two research team members (SLP and MG) independently read the transcripts and developed codes as well as themes. The 2 codebooks were compared for congruency before all transcripts were coded. Through this process, themes and principles emerged that characterized participants’ priorities.

## 3 RESULTS

Table 2 shows voting results of the 5 strategies to reduce overuse and how votes were associated with the case scenarios. This table is a summary of a more detailed version of participants’ voting (see Supporting Information B). Participants’ discussion of overuse, the possible strategies to address the problem and their role in influencing health‐care policy generated 5 predominant themes.

**Table 2:** Doing What Works: public deliberation on the best strategy for reducing overuse in medical care (N = 117)

Case scenarios	Five strategies to reduce overuse				
	Establish greater physician oversight (%)	Influence physicians through financial rewards (%)	Influence physicians through reduced payment (%)	Increase patient cost‐sharing for low‐value care (%)	Take no action: continue as a doctor–patient decision only (%)
1. Antibiotics: what is the best way to discourage overuse of antibiotics?	82	4	(not available)	30	17
2. Caesarean births: what should be performed to encourage proper use of C‐section?	72	1	43	54	15
3. MRIs: what should be performed to encourage proper use of MRIs for low‐back pain?	100	(not available)	10	7	10
Total votes for each strategy	254	5	53	91	42
Percentage of participants who chose this^a^	72%	2%	23%	26%	12%

Authors’ analysis of data from Doing What Works deliberation sessions, 2015.

a The percentages are determined by the number of votes for this strategy divided by the number of potential votes (number of participants, 117, times the number of scenarios, either 2 or 3, where the strategy was available.) The total percentages sum to greater than 100% because participants could choose more than one strategy for each case scenario.

### 3.1 Strategies to reduce overuse

#### 3.1.1 Physician leaders are responsible for resolving the overuse problem

Across all 3 scenarios, monitoring of physicians by physicians was chosen by 72% of the participants, far more often than the other strategies. The key rationale is that physicians must be in control of instituting their own corrective actions. Although participants were concerned about administrative burden, few doubted that the task of reducing overuse rested with the medical profession. Participants were willing to overlook the administrative burden *if* corrective action is the most effective, retrospective review is a fairer approach than pre‐approvals, and physicians were willing to judge and be judged by each other.

#### 3.1.2 Monetary incentives are inconsistent with medical professionalism

Only 5 participants supported the option of rewarding physicians for reducing overuse (a strategy offered in 2 of the scenarios), representing 2% of the total votes. There was almost universal opposition to paying doctors more for “doing what they should be doing.”

Many saw this approach as contrary to medical professionalism that purports to embrace standards of excellence and allegiance to patient well‐being and not the self‐interests of practitioners.^16^ Rewarding doctors to improve their performance seemed demeaning for a profession the participants hold in such high regard. Participants believed that doing their best for their patients, not increasing their income, must motivate doctors.

Interestingly, with 23% of the votes, participants were more accepting of the strategy not to compensate doctors for chronic overuse. While most felt strongly that physicians’ treatment decisions should not be tied to compensation in any way, the Caesarean birth example indicated that the overuse problem in California is largely driven by physicians and hospitals and not by patients. It appears that this fact (and the cost and number of unnecessary procedures) elicited atypical enthusiasm for denying provider payments. However, the majority still preferred using strategies that relied on medical professionalism.

#### 3.1.3 Higher patient cost‐sharing may be justified to maintain freedom of choice

The option to increase patient cost‐sharing for unnecessary interventions was proposed in all 3 scenarios. While the total support for this strategy was just 26%, it was most favoured in the Caesarean birth example, where almost half of all participants supported it.

Many participants regard childbirth as a health‐care domain that is distinctive from others, where a women’s right to make decisions about her own body is almost sacrosanct. In this situation, their strong belief in patient choice was balanced by their conviction that the state should not waste its resources on unnecessary care. They believed that higher patient cost‐sharing would dampen enthusiasm for the procedure while maintaining a woman's right to choose it. Contrary to research,^17^ many participants believed the high Caesarean birth rate was a result of patient demand. Participants who objected to this strategy believed it was unethical for physicians to provide potentially harmful services, regardless of who paid for it.

#### 3.1.4 Responsible use of shared resources dominated the discussions

Although each case scenario emphasized medical harms (individual and, at times, societal), participants tended to focus almost exclusively on the waste of communal resources as a motivator for action. These low‐to‐moderate income Californians seemed acutely aware of increased health‐care costs and the impact this has on the services they receive. While many were aware of and alarmed by the problem of antibiotic resistance and its impact on society at large, the statistics associated with the individual harms of unnecessary Caesarean births and MRIs did not resonate with participants.

Their concern about wasting societal resources, however, was not limited to overuse, per se. The strategies that are intrusions into independent doctor‐patient decision‐making must be justified by evidence that these strategies are, in fact, effective in reducing waste.

#### 3.1.5 The citizen voice is not the same as the patient voice

Participants made it clear that their views reflected the role they were asked to assume as follows: that of policymaker, not of patient. Many stated, “ if I am responding as a patient, I would take no action and continue to leave it to the doctor and patient to decide.” Conversely, they acknowledged that as a citizen decision maker, they were responsible to many more people and most endorsed actions that might, in fact, hamper the doctor‐patient autonomy that they, as patients, preferred.

There was considerable discussion on the need for more education to help both patients and the general public fully understand the problems of overuse. Educating patients in the context of their own medical care must be matched by a broader societal emphasis on financial and medical harm.

## 4 DISCUSSION

### 4.1 Implications for policymakers

In reviewing the *DWW* results and themes that emerged, health‐care leaders were especially interested in how the findings are congruent with and/or depart from existing strategies to reduce overuse. As Smart Care California develops and implements approaches to reducing overuse, the *DWW* findings can help inform their activities and decision‐making. The following describes some of the overuse strategies now being used.

#### 4.1.1 Reduce overuse through physician‐led efforts

As noted earlier, participants regard these clinical decisions as ones “belonging to the profession” and thus, it is the profession that must remedy problems among its members.

This perspective is consistent with and widely endorsed by state and national initiatives.^18^, ^19^, ^20^ Most visible is Choosing Wisely^®^, a national programme sponsored by the American Board of Internal Medicine Foundation in partnership with more than 75 national medical societies.^21^ CW believes that changing physician practice must be grounded in professionalism with actions that the profession itself must determine and control; it rejects the policy of non‐payment or lowering compensation to change practice patterns.^21^ Nationally, the launch of CW has modestly decreased overuse of key low‐value services.^22^

Hospitals in California have initiated programmes that focus on physicians working with their colleagues to improve practices.^10^ For example, Cedars‐Sinai Health System incorporated 26 of the CW specialty society campaign recommendations into its electronic medical records system using alerts to physicians when they order an overused intervention. The number of electronic medical record alerts specific to unnecessary imaging was significantly reduced over an 18‐month period and they estimate that together all CW alerts saved their health system $200,754 in unnecessary services over a 6‐month period.^23^

In an effort to broaden CW initiatives, 2 medical groups, a physician organization and a consumer group collaborated to integrate CW recommendations into clinical practice. These medical groups began tracking and reporting site‐level change to clinicians over time on imaging for uncomplicated headache to provide comparative performance feedback.^24^

#### 4.1.2 Influence physician practices through reduced compensation

*DWW* participants only marginally supported reducing compensation for physicians who consistently prescribe low‐value care, but were more inclined to do so for unnecessary C‐sections.

In California, hospitals are experimenting with the use of monetary disincentives with notable success. To remove financial incentives for Caesarean births, 3 hospitals negotiated a blended case rate for deliveries—one flat rate regardless of delivery method (Caesarean or vaginal). These hospitals concurrently implemented processes for data and measurement of physician‐level Caesarean rates and a quality improvement programme. Using this three‐pronged approach—payment reform, quality improvement and physician feedback—the average number of Nulliparous, Term, Singleton, Vertex (NTSV) Caesarean births was reduced by 20% in less than 1 year.^25^

#### 4.1.3 Influence physician practices through increased compensation

Of all the strategies proposed, this one elicited the strongest opposition from *DWW* participants with only 2% of participants supporting this option.

Ironically, California has been a long‐standing leader in Pay‐for‐Performance (“P4P”) programmes that acknowledge and reward physician groups showing improvement in quality of care measures.^26^ While California's P4P programme shows mixed results—demonstrating incremental, not breakthrough, gains in quality measure outcomes over time—physician organizations agree that this programme has forced an alignment of measure sets across health plans and has created a positive competitive incentive among physician organizations.^27^

Within the P4P programme in California, Hill Physicians Medical Group, a very large Northern California Independent Practice Association, has—for more than 15 years—been using financial incentives that now amount to over 30% of primary care physicians’ compensation. Of the dozen quality measures most recently in its primary care incentive programme, one focused explicitly on overuse. This measure, avoiding overuse of antibiotics for acute bronchitis, showed a solid 8.5% improvement in 2016 compared to 2015. The quality incentive programme has proven effective in moving from volume to value, it pays for both attainments against external benchmarks as well as improvement towards those benchmarks. (David Joyner, CEO Hill Physicians Medical Group, personal correspondence, April 11, 2017)

The jury may still be out on whether physician practices can be meaningfully influenced through professionalism alone or whether financial motivation—sticks or carrots—is needed.

#### 4.1.4 Reduce patient demand by increasing patient cost‐sharing for unnecessary care

With 26% of the votes, this strategy was not a dominant one in *DWW*. Those who supported it did so because it retained patient authority, even over ineffective and harmful medical treatment. Patient choice is a long‐standing, well‐engrained value in American health care.^28^

California policymakers have indicated interest in ways to reduce patient demand for unnecessary care^10^ but to date, the practice of charging patients more for low‐value care has been used sparingly.^29^ CalPERS has implemented, with measurable success, a method called reference pricing, where patients pay more if they choose a medical service that is more expensive than one of comparable quality.^30^ This strategy is specific to prescribed interventions with considerable cost variations. Recently, some health plans have introduced higher cost‐sharing when patients use certain services unnecessarily, such as using the Emergency Department without sufficient cause.^31^ But both these examples are ones where the patient is the seeker of services; the physician is not making a treatment decision or choosing where and how the patient seeks a treatment. Thus, this increase in cost‐sharing is less controversial and less complex to employ than in situations where treatments must be authorized by physicians. Some state programmes have tied higher cost‐sharing to preference‐sensitive interventions,^32^ a strategy that is trying to orient cost‐sharing to interventions of questionable clinical value.

#### 4.1.5 Increase the visibility of low‐value care, its harms and costs

Participants conveyed their strong support for disseminating more information to patients and to consumers in general about the harms and costs of unnecessary care.

There is a growing volume of patient materials about the harms associated with the use of unnecessary medical care. Although CW has included some information in its patient‐facing materials about wasting resources,^21^ this has not been a widely used communication campaign among the general public. Smart Care California and consumer advocacy groups might consider ways to make this problem more visible to the public. The California Medical Association has developed materials as a part of their Antibiotic Resistance Education (AWARE) multistakeholder campaign in 2000 to promote the appropriate use of antibiotics. At a minimum, communications strategies should be tested with consumers.

Individual patients are not likely to respond positively to discussions of financial harm or resource stewardship. However, when people learn about the impact of the financial harms of overuse on the health‐care system outside their interactions with their health‐care providers, they are more likely to view the problem from a purchaser, co‐payer or citizen role.^33^

#### 4.1.6 Assure that the citizen voice has a role to play in policy changes

*DWW* participants embraced this role, even while acknowledging that it may conflict with their views as patients.

This contrast between the perspectives of the patient and those of the informed citizen is also evident in survey research.^14^ When asked to respond as a patient (*If my doctor and I agree on the best treatment for my problem, my health plan should pay for it no matter what the research shows*), 65% of participants agreed with that statement. But when the question was asked indirectly (*Health plans should pay for any treatments that doctors recommend, even if research shows that a treatment does not work well for patients*), only 27% agreed.^14^ Individuals feel strongly that their voice is important to bring to policymakers with 91% indicating that “it is very important that health‐care leaders understand the views of people like me.”^14^

Health‐care policymaking typically involves 4 major groups: purchasers, health plans, regulators/legislators and providers. The findings from *DWW* suggest value in including another stakeholder group: the informed citizen. Just as purchasers and providers have different priorities and perspectives, *DWW* illustrates that citizens approach the problem of overuse from distinct vantage points.

### 4.2 Limitations

The sample size of 117 lower‐to‐moderate income California residents does not necessarily represent the views and values of the state's population at large. The overrepresentation of females does not reflect the population, but it is common and often expected in social research studies.”^34^ The problem of overuse is complex and longer deliberation on the topics of evidence‐based medicine and the meaning of value‐based health care might generate different results.

## 5 CONCLUSION

Most of the perspectives voiced by the *DWW* participants are congruent with efforts to reduce overuse that are being initiated at the state, provider and health plan level. The public's disapproval of programmes that reward doctors for good quality care suggests that consumer‐facing communications should focus on recognition of excellence rather than monetary rewards. Increasing patient cost‐sharing to maintain patient choice without jeopardizing shared resources is appealing to many but not feasible to implement in Medicaid. The lay public also shares the concern of many medical professionals on the ethics of prescribing unneeded, potentially harmful care, regardless of who is paying.

Civic deliberation of complex health‐care topics is not a common practice in the US. Yet, as long as health‐care reform continues to focus on cost containment policies, the public voice will play an important role in balancing the tension between cost and benefit. Harmonization of evidence‐based practices, responsible use of resources and patient preference is not easily achieved. Future research on public values might explore more closely how to best reconcile these often‐conflicting values.

## COMPETING INTERESTS

None declared.

## Note

1 All Covered California and CalPERS participants had an annual income below 400% of the Federal Poverty Level (FPL), and all Medi‐Cal participants had an annual income below 138% of the FPL—a requirement to qualify for this program.
