# Supplementary material for: Assessing social values for California's efforts to reduce the overuse of unnecessary medical care
Source: Health Expect. 2017 Nov 16;21(2):501–7. doi: 10.1111/hex.12644 (PMC5867318; doi:10.1111/hex.12644)
Supplement: Supplementary file 1 [file HEX-21-501-s001.docx]

Using antibiotics for adult bronchitis

Antibiotics

Antibiotics are drugs used to control infections that are caused by bacteria. They are often a life-saving treatment for patients with deadly infections. Over the years, different antibiotics have been developed for a variety of bacterial infections. Because they are easy to use and most people have had them, antibiotics have become a familiar treatment. However, the most common types of infections among children and adults – such as colds, the flu, and ear infections – are caused by viruses, not by bacteria. Antibiotics do not kill viruses; they only work when bacteria cause the sickness.

Adult bronchitis

Acute bronchitis (chest cold) is one of the most common reasons that adults go to the doctor. Bronchitis usually includes a cough, lasts no longer than three weeks, and is caused by a virus. Although antibiotics are not useful for bronchitis, doctors order them frequently for their patients. In fact, a recent study showed that up to 71% of patients with acute bronchitis are getting antibiotics from their doctors.

The harm of over-use

When antibiotics are given a lot, bacteria can become “resistant.” Antibiotics do not work well at killing these resistant bacteria. Over-use brings harm in three ways:

*Greater risk to the individual.* Antibiotics can have harmful side effects, ones that are sometimes dangerous for patients. Also, if a patient has antibiotics often, she or he may be more likely to get sick from resistant bacteria. This puts the patient in greater danger of having an infection that cannot be controlled.

*Puts others at risk.* When antibiotics are over-used, super-resistant bacteria (a “super-bug”) may develop that no antibiotic can kill. This means that patients everywhere may risk an infection that cannot be treated. These super-bugs now sicken 2 million Americans each year and kill 23,000 people.

*Greater cost to society.* Although many antibiotics are not expensive, treating patients who are extremely ill with an uncontrolled infection adds to the cost of health insurance for everyone. For example, patients in the hospital with resistant bacteria must stay in the hospital twice as long as patients who do not have infections.

Expert medical groups have published guidelines for many years, warning doctors about over-use. Despite these guidelines, the problem continues.

C-sections with normal pregnancies

Importance of C-sections

Cesarean birth (also called C-section) is when a baby is delivered through surgery into the mother’s belly and womb. C-sections are medically necessary when a vaginal delivery may be dangerous for the mom – or when the baby is having a problem that requires a fast delivery. Although most women have healthy, normal pregnancies, C-sections are an important life-saving method for the mothers and their babies who need one.

As doctors and hospitals have become more skilled, problems from this surgery – such as infections or harm to the child– have dropped. Since then, the number of C-sections has grown by 50% in California. Now, 1 in every 3 births is done by C-section.

Why are there more C-sections now?

Medical experts believe that no more than 15% of deliveries should require a C-section. Yet in California, the C-section rate varies from 13% to 83%, depending on the hospital. But there is no difference in the health of the mothers or babies that would explain these higher numbers.

Researchers learned that these higher C-section rates are not because women or babies have more medical problems than before; or because women are asking for C-sections; or because of doctors’ fear of lawsuits. Rather, C-sections are more common because:

It is more convenient for doctors and hospitals to schedule C-sections.

C-sections reduce the staff time needed to support women during childbirth.

Hospitals and doctors receive higher payments for C-sections (about $9,000 more).

Americans have become more casual about surgery and do not understand the possible risks it brings.

Yet, there is no medical benefit for women or babies when C-sections are done without a medical reason. But there is greater harm.

The harms of unnecessary C-sections

Childbirth is the #1 reason for being in the hospital, and there are 500,000 births in California each year. So the over-use of C-sections has a big impact.

*Medical harm*. Although safer now, C-sections usually require a longer recovery time, and increase the chance of infection, excessive bleeding, and postpartum depression. And babies are at slightly higher risk of developing diseases like asthma and diabetes.

*Financial harm*. For those with private health insurance, the higher cost of C-sections means that the mother and her health plan will both be paying more than is necessary.

*Societal harm.* Medi-Cal pays for half of the child-births in California. Spending more on unnecessary services means there is less money in the Medi-Cal budget for high-value services or new benefits.

Using MRI scans for low back pain

What is an MRI?

An MRI is a “high-tech” scanning machine that lets doctors see inside parts of the body, such as organs, bones and blood vessels. This works well to help doctors identify certain medical conditions – such as tumors or problems with joints or blood flow – and choose the treatment.

Since MRIs were invented in the 1980s, the number of MRI machines in the U.S. has grown greatly. This makes it easy for doctors to order more MRI scans than in earlier years. Medical researchers have studied when MRIs are useful in diagnosing a patient’s condition and when they are not useful.

Low back pain

Eight of every 10 American adults will have had low back pain at some time in their life. It can happen from heavy lifting, chores, a sports injury, or just a twist the wrong way. It usually doesn’t last long, but it can greatly limit everyday activities. The doctor will examine the patient and, if there are no worrisome signs, may suggest physical therapy (PT), heat and medicines to reduce pain. It usually takes 2-6 weeks for the back pain to stop. If there are signs that it might be a more serious problem, then the doctor orders other tests, possibly an MRI. But for common low back pain, an MRI will not show anything helpful for treating the patient. Despite medical guidelines on the correct use of MRIs, many doctors order MRIs even when there is no sign that the patient needs one.

The harm of over-use

*Medical harm to the patient.* The scan may show something unusual that isn’t really a medical problem. This can lead to surgery that the patient didn’t need. In fact, researchers have shown that when scans are done without a strong medical reason, patients are 8 times more likely to get surgery that was not needed. These patients had no better results from their back surgery than those who did not get surgery. And surgery itself exposes people to many possible harms.

*Financial harm for others*. One research report showed that only a third of all lower back MRI’s were considered necessary. Nationally, the overuse of all scans and x-rays means there is as much as $30 billion in unnecessary spending.

But like the overuse of antibiotics, simply educating doctors and patients about this does not change what they do!
