# Supplementary material for: Assessing social values for California's efforts to reduce the overuse of unnecessary medical care
Source: Health Expect. 2017 Nov 16;21(2):501–7. doi: 10.1111/hex.12644 (PMC5867318; doi:10.1111/hex.12644)
Supplement: Supplementary file 2 [file HEX-21-501-s002.docx]

| ***Doing What Works:* Participants’ responses to the options offered for each case scenario and the types of strategies they represent** | | | | | |
| --- | --- | --- | --- | --- | --- |
|  | Five types of strategies | | | | |
| **Case Scenarios and their options for**  **reducing overuse** | **Establish greater physician oversight** | **Influence physicians through rewards** | **Influence physicians through reduced payment** | **Increase patient cost-sharing for low-value care** | **Take no action: continue as a doctor/ patient decision only** |
| ANTIBIOTICS: What is the best way to discourage over-use of antibiotics? |  |  |  |  |  |
| 1. If a doctor routinely over-uses antibiotics, that doctor should have to get approval from a medical expert before ordering one. | 40 |  |  |  |  |
| 2. To discourage patients from insisting on an antibiotic, the patient should have to pay most of the cost of the drug if the doctor cannot give a good medical reason for ordering it. |  |  |  | 30 |  |
| 3. Doctors that work together should monitor each other. If any of them continue to over-use antibiotics, they could be disciplines in some way. | 42 |  |  |  |  |
| 4. To encourage doctors to follow guidelines, they should be rewarded with a small bonus if they are careful with antibiotics. |  | 4 |  |  |  |
| 5. Continue to leave it to the patient and doctor to decide if an antibiotic is needed or not, despite the risks. |  |  |  |  | 17 |
| CESAREAN BIRTHS: What should be done to encourage proper use of C-section? |  |  |  |  |  |
| 1. Monitor doctor’s practices; health plans would provide a small bonus for those who follow guidelines for doing C-sections. |  | 1 |  |  |  |
| 2. Encourage pregnant women to choose vaginal births (when there is no medical problem) by offering a valuable gift (for example, a gift card). |  |  |  | 3 |  |
| 3. Require all scheduled C-sections to be reviewed and approved in advance by expert doctors that have no financial motives. | 72 |  |  |  |  |
| 4. Give health plans the authority to reduce payment to a doctor who, based on a review by an independent panel, performed an unnecessary C-section. |  |  | 43 |  |  |
| 5. Continue to leave it to the patient and doctor to decide if an antibiotic is needed or not, despite the risks. |  |  |  |  | 17 |
| 6. (Additional option proposed by six groups) If a patient wants an unnecessary C-section, she pays the extra cost. |  |  |  | 54 |  |
| MRIs: What should be done to encourage proper use of MRIs for low back pain? |  |  |  |  |  |
| 1. Doctors who often over-use MRIs without good medical reasons should be required to first get approval from a medical expert before ordering an MRI. | 24 |  |  |  |  |
| 2. If a doctor orders an MRI without a strong medical reason, the doctor should have to pay the cost of it. |  |  | 10 |  |  |
| 3. To encourage patients to think twice before insisting on an MRI when there is no medical reason, the patient should have to pay the cost of it. |  |  |  | 7 |  |
| 4. There should be stricter rules for when an MRI can be ordered for common low back pain. For example, it could be ordered only if the back pain is not better in 4-6 weeks. | 76 |  |  |  |  |
| 5. Continue to leave it to the patient and doctor to decide if an MRI is needed or not, regardless of the possible harm. |  |  |  |  | 10 |
| **TOTAL VOTES for each strategy** | **254** | **5** | **53** | **91** | **42** |
| **Percent of participants who chose this*** | **72%** | **2%** | **23%** | **26%** | **12%** |
| * The percentages are determined by the number of votes for this strategy divided by the number of potential votes (number of participants, 117, times the number of scenarios, either two or three, where the strategy was available.) The total percentages are greater than 100% because participants could choose more than one strategy for each case scenario. | | | | | |
